# Optical scatter patterns facilitate rapid differentiation of *E* *nterobacteriaceae* on CHROMagar^TM^ Orientation medium

**DOI:** 10.1111/1751-7915.12323

**Published:** 2015-10-27

**Authors:** Atul K. Singh, Arun K. Bhunia

**Affiliations:** ^1^ Department of Food ScienceMolecular Food Microbiology LaboratoryPurdue UniversityWest LafayetteIN47907USA; ^2^ Department of Comparative PathobiologyPurdue UniversityWest LafayetteIN47907USA

## Abstract

*E* *nterobacteriaceae* family comprised pathogens and commensals and has a significant impact on food safety and public health. *E* *nterobacteriaceae* is often enumerated and presumptively identified on chromogenic media, such as CHROMagar^TM^ Orientation medium based on colony profile; however, classification is highly arbitrary, and some could not be differentiated due to similar chromogen production. Here, we investigated the ability of the laser optical sensor, BARDOT (bacterial rapid detection using optical scattering technology) for rapid screening and differentiation of colonies of the major bacterial genera from *E* *nterobacteriaceae* on CHROMagar^TM^ Orientation. A total of 36 strains representing 12 genera and 15 species were used to generate colony scatter image library that comprised 1683 scatter images. This library was used to differentiate mixed cultures of *E* *nterobacteriaceae* family – *K* *lebsiella pneumoniae*, *E* *nterobacter* spp., *C* *itrobacter freundii* and *S* *erratia marcescens* (KECS group); *P* *roteus mirabilis*, *M* *organella morganii* and *P* *rovidencia rettgeri* (PMP group); and non‐*E* *nterobacteriaceae* family: *P* *seudomonas aeruginosa*, *A* *cinetobacter* spp. and *S* *taphylococcus aureus* (PAS group) – and data show high accuracy (83–100%) for intra‐group classification of colonies in 10–22 h or even before visible production of chromogens. BARDOT successfully differentiated the major genera, including the ones that do not produce visually distinguishable chromogens on CHROMagar^TM^ Orientation, providing a label‐free, real‐time on‐plate colony screening tool for *E* *nterobacteriaceae*.

## Introduction

*Enterobacteriaceae* (EB) is the largest family (∼ 47 genera and ∼ 221 species) in bacterial taxonomy, which comprised enteric pathogens, food‐ and water‐borne pathogens, uropathogens, and commensals (Baylis *et al*., 2011; Temkin *et al*., 2014). *Enterobacteriaceae* is also a major public health concern due to its involvement in community‐acquired and nosocomial diseases, and its resistance to β‐lactam and carbapenem group of antibiotics (Zurfluh *et al*., 2013; Temkin *et al*., 2014). In the food industry, EB has been widely used as an indicator microorganism to evaluate microbiological quality and safety, and to assess sanitary and hygienic practices employed during food production, preparation, handling and storage (Stedtfeld *et al*., 2007; Kornacki, 2011; Buchanan and Oni, 2012; Cerna‐Cortes *et al*., 2012; Holvoet *et al*., 2014; Barco *et al*., 2015).

Emerging detection and diagnostic technologies, such as those based on biosensors, together with the routine microbiology techniques could expedite detection, diagnosis and therapeutic intervention (Berlutti *et al*., 1989; Liao *et al*., 2006; Owen *et al*., 2010; Velusamy *et al*., 2010; Xu *et al*., 2012; Clark *et al*., 2013; Bhunia, 2014; Buchan and Ledeboer, 2014; Hasman *et al*., 2014). With the new challenges and solutions for modern clinical microbiology, the term ‘culturomics’ has been proposed to emphasize the diversification of culture condition for improved isolation of fastidious microorganisms (Fournier *et al*., 2013). Furthermore, culturomics could be integrated with the evolving next‐generation molecular, immunological, nanobiotechnological and biophysical assays for identification of pathogens in diagnostic laboratories (Fournier *et al*., 2013).

BBL^TM^ CHROMagar^TM^ Orientation medium (BD, Franklin Lakes, NJ) is a non‐selective differential medium that has facilitated rapid identification of colonies of the majority of pathogens in *Enterobacteriaceae* (Samra *et al*., 1998; Chaux *et al*., 2002; D'Souza *et al*., 2004; Manickam *et al*., 2013; Payne and Roscoe, 2015). For example, species of *Klebsiella*, *Enterobacter*, *Citrobacter* and *Serratia* (KECS group) produce metallic/turquoise blue colonies; *Proteus*, *Morganella* and *Providencia* (PMP group) produce translucent colonies with brown halo, while some non‐*Enterobacteriaceae*, *Pseudomonas*, *Acinetobacter* and *Staphylococcus* (PAS group) produce cream‐coloured colonies (Samra *et al*., 1998; Chaux *et al*., 2002; D'Souza *et al*., 2004; Payne and Roscoe, 2015). However, the major limitation is that the chromogen alone cannot distinguish among the colonies of KECS, PMP and PAS groups. Thus, additional alternative tests are needed for microbial differentiation within a group.

Previously, our group has developed a non‐invasive laser optical sensor, BARDOT (**b**acterial **r**apid **d**etection using **o**ptical scattering **t**echnology) that can differentiate colonies of bacteria at the genus, species and serovar levels based on colony scatter signatures without using any biological or chemical probes (Bhunia, 2008; 2014). BARDOT is equipped with a red diode laser (635 nm, 1 mW, 1 mm diameter beam), and when the laser shines on the centre of a colony, it instantly generates a forward scatter image captured by a CCD camera (Banada *et al*., 2007). Such optical sensors generally work on the principles of the physics of diffraction, interference and refraction when the laser beam passes through the centre of individual colony (Bae *et al*., 2007). The identity of the colony is interrogated upon matching the scatter image with a pre‐established scatter image library using the image classifier (Ahmed *et al*., 2013). We have successfully used this sensor for detection of *Listeria* species, including *L. monocytogenes* (Banada *et al*., 2007; 2009), *Salmonella enterica* (Singh *et al*., 2014), Shiga‐toxigenic *E. coli* (Tang *et al*., 2014), *Vibrio* spp. (Huff *et al*., 2012), *Campylobacter* spp. (He *et al*., 2015) and *Bacillus* spp. (Kim *et al*., 2014; Singh *et al*., 2015b) directly on Petri plates. Recently, we also applied BARDOT to study the streptomycin‐induced stress response in *Salmonella enterica* serovar Typhimurium based on differential colony scatter patterns (Singh *et al*., 2015a). In addition, it was also used as a bioanalytical detection tool to validate performance of a sample processing and enrichment device, a pathogen enrichment device (Hahm *et al*., 2015).

**Figure 1:**
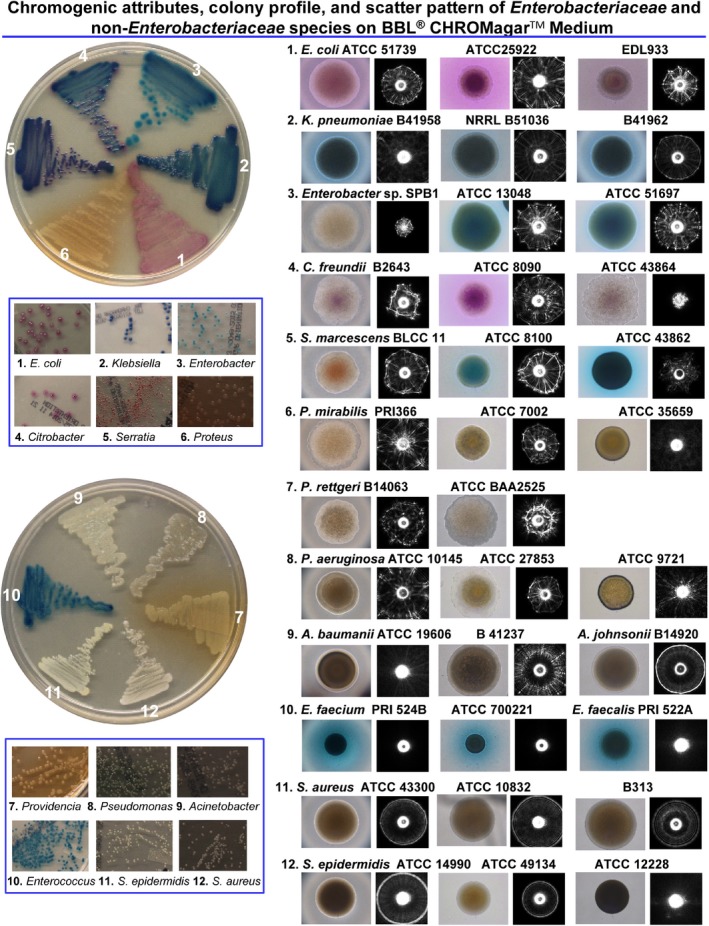
Bacterial colony colour and scatter pattern on CHROMagar^TM^ Orientation medium after 10–22 h of growth. Streaked plate image: images of single colonies in rectangular panel depict chromogenic attributes after 24–48 h of incubation; single colony microscopic images indicated colony profile as well as chromogenic attributes after 10–12 h of incubation; black and white images are the scatter pattern of single colony. Colony profile was observed after phase contrast microscopy of ∼ 1 mm diameter colony at 100× magnification. Bacterial cultures were grown in BHI broth at 37°C overnight (14–16 h), serially 10‐fold diluted in 100 mM PBS (pH 7.4), and were plated on readymade BBL^TM^ CHROMagar^TM^ Orientation medium (BD) to obtain 50–100 colonies/plate. The scatter patterns of bacterial colony were captured using BARDOT when the colony diameter reached to 1.0 ± 0.2 mm in about 10–22 h. Plate picture and its inset were taken at ∼ 24 h of incubation to confirm the chromogenic attributes of cultures on CHROMagar^TM^ Orientation medium. The appearance of expected colour of colony on the CHROMagar^TM^ Orientation medium took minimum 24–48 h, whereas some of the tested species/strains (*E* *nterobacter* sp. SPB1; *C* *. freundii* B2643, ATCC 8090, and ATCC 43864; *S* *. marcescens* BLCC11) did not show the expected colour at 10–22 h when the scatter pattern of colonies (1.0 ± 0.2 mm diameter) were captured.

Here, we investigated whether BARDOT could be used for high‐throughput screening and detection of colonies of the members of *Enterobacteriaceae* on a commonly used CHROMagar^TM^ Orientation plate based on scatter pattern. BARDOT‐generated scatter pattern could be used to differentiate colonies of *Enterobacteriaceae* and a few non‐*Enterobacteriaceae* within the KECS, PMP and PAS groups, which cannot be otherwise differentiated due to production of similar chromogens on CHROMagar^TM^ Orientation medium (Fig. 1). The results of this study will aid clinicians to make rapid diagnosis of pathogens for specific therapeutic intervention at the point of care, and food microbiologists to make a rapid assessment of microbiological safety/quality of the products.

## Results and discussion

### Chromogenic attributes and scatter image library

A total of 36 strains of *Enterobacteriaceae* and non‐*Enterobacteriaceae* genera representing 12 genera and 15 species were used in this study (Table 1). The scatter pattern of colonies on plates were acquired by BARDOT when the colony diameter reached 1.0 ± 0.2 mm, taking about 10–22 h of incubation for the majority of tested strains with the exception of a few strains that took around 36 h to reach the desired colony size (Table 1). A general *Enterobacteriaceae* (EB) and non‐*Enterobacteriaceae* (NE) scatter image library designated EBNE‐11, representing 11 genera, 14 species (3 strains per species with the exception of *Morganella* and *Providencia*) (Table 1) was built. The EBNE‐11 library consisted of a total of 1683 scatter images representing minimum 50 images per strain obtained from two independent experiments with two technical replicates (*n* = 4) to account for any strain‐to‐strain variations.

**Table 1:** List of bacterial cultures used in this study and their characteristics and classification accuracy on CHROMagar^TM^ Orientation medium

*Bacteria*	Strain^a^	Colony colour on CHROMagar™ Orientation	Incubation time (h) for colonies to reach 1.0 ± 0.2 mm diameter	% Average positive predictive values (PPVs) ± SEM
*Enterobacteriaceae*				
*Escherichia coli*	ATCC 51739, ATCC BAA 25922, EDL933	Pink	12–17 h	83.4 ± 3.5
*Klebsiella pneumoniae*	B41958, B51036, B41962	Metallic blue	12–17 h	74.9 ± 4.9
*Enterobacter* spp.	SPB1, ATCC 13048, ATCC 51697	Metallic/Turquoise blue	10–11 h	90.1 ± 1.5
*Citrobacter freundii*	NRRL B2643, ATCC 8090, ATCC 43864	Metallic blue with red halo	10–17 h	81.9 ± 0.1
*Serratia marcescens*	BLCC 11, ATCC 8100, ATCC 43862	Metallic blue	12–19 h	74.2 ± 1.5
*Proteus mirabilis*	PRI 366, ATCC 7002, ATCC 35659	Brown halo	13–21 h	77.6 ± 1.2
*Providencia rettgeri*	B14063, ATCC BAA 2525	Brown halo	11–36 h	92.2 ± 1.0
*Morganella morganii* ^b^	ATCC 25830	Translucent, cream	27 h	Not analysed
Non‐*Enterobacteriaceae*				
*Pseudomonas aeruginosa*	ATCC 10145, ATCC 27853, ATCC 9721	Translucent, cream	14 h	94.0 ± 0.6
*Acinetobacter baumannii/johnsonii*	ATCC 19606, B41237, B14920	Cream	10–22 h	87.7 ± 3.2
*Enterococcus faecium*/*aerogenes* ^b^	PRI 524B, ATCC 700221, PRI522A	Turquoise blue	17–36 h	Not analysed
*Staphylococcus aureus*	ATCC 43300, ATCC 10832, NRRL B313	Golden, opaque	13–17 h	97.8 ± 0.6
*Staphylococcus epidermidis*	ATCC 14990, ATCC 49461, ATCC 12228	Cream, pinpoint	19–36 h	96.6 ± 0.3

**a**. Total 36 strains representing 12 genera and 15 species were used in this study to capture the scatter pattern (1683 images) on CHROMagar^TM^ Orientation medium. Cultures were procured from ATCC, American Type Culture Collection, Manassas, VA; BLCC, Bhunia Lab Culture Collection, Purdue University, West Lafayette, IN; Presque Isle Cultures, Erie, PA; NRRL, Northern Regional Research Laboratory, Peoria, IL. EDL strain was obtained from CDC, USA. *Enterobacter* sp. SPB1 is a spinach isolate from our previous study (Singh *et al*., 2014). **b**. Scatter patterns of *Morganella morganii* were not included in the EBNE‐11 library since it consisted of only one strain. Scatter images of *Enterococcus* spp. were also not used in the analysis, since the sensor could not capture adequate numbers of scatter patterns in an automated mode due to the opacity of colonies. A few images for this genera (total 4) were captured in the manual mode (Fig. 1). PPVs are represented as % average PPV ± standard error of mean (SEM) values calculated from the scatter images acquired from two independent experiments with two replicates (*n* = 4).

Since, the BARDOT system works on the principle of forward light scattering, the incident laser should be able to pass through the colonies on agar media, so that the scatter images are captured by a camera placed beneath the agar plate. Opaque colonies with excessive chromogens may hinder laser propagation, thus may not generate scatter patterns. To overcome such limitation, we are now modifying the BARDOT system to capture the backscatters for colony identification.

After performing image analysis using EBNE‐11 library, the accuracy of differentiation, also expressed as positive predictive value (PPV), was estimated to vary from 81.9 ± 0.1% to 97.8 ± 0.6% (Fig. 1, Table 1), while the PPVs for the remaining three species – *S. marcescens* (74.2%), *K. pneumoniae* (74.9%) and *P. mirabilis* (77.6%) – were each very low (Table 1). This variation in PPVs can be attributed to the overlapping scatter features (Zernike moments, ring and spoke patterns; Haralick textures, image texture) among the test strains. In order to improve our overall classification accuracy or to obtain higher PPV for a species within EB, especially among the pathogens within a group that cannot be differentiated based on the colour alone on CHROMagar^TM^ Orientation medium, separate scatter image libraries were built using scatter images from the EBNE‐11 library (i) ‘KECS‐Library’ representing *Klebsiella*, *Enterobacter*, *Citrobacter* and *Serratia*; (ii) ‘PMP‐Library’ representing *Proteus*, *Morganella* and *Providencia*; and (iii) ‘PAS‐Library’ representing *Pseudomonas*, *Acinetobacter* and *Staphylococcus* (Table 2). We have previously shown that appropriate pathogen(s)‐specific libraries can be used for specific identification of an organism. For example, *Salmonella* spp. was detected on XLT‐4 agar plate in the presence of background microbiota (*E. coli* and *Enterobacter* spp.) from chicken and fresh produce (Singh *et al*., 2014), and *Bacillus* spp. was detected on phenol red mannitol plate in the presence of background microbiota (*Staphylococcus*, *Enterococcus*, *Micrococcus* and *Serratia* genera) from bovine raw milk (Singh *et al*., 2015a).

**Table 2:** Analysis of scatter pattern on CHROMagar^TM^ Orientation medium of colonies of *E* *nterobacteriaceae* family and few non‐*E* *nterobacteriaceae* species

Bacteria	Strains tested (*n*)	% Average scores^b^ ± SEM			
Scatter image library^a^		Sensitivity	Specificity	Negative predictive value (NPV)	Positive predictive value (PPV)
*Enterobacteriaceae*					
KECS‐group					
*Klebsiella pneumoniae*	3	87.1 ± 0.1	73.1 ± 0.1	90.1 ± 0.5	88.2 ± 2.2
*Enterobacter* spp.	3	93.2 ± 0.8	97.6 ± 0.3	88.2 ± 1.0	93.8 ± 1.0
*Citrobacter freundii*	3	90.1 ± 0.8	97.4 ± 0.1	88.4 ± 1.2	93.2 ± 0.3
*Serratia marcescens*	3	88.7 ± 1.6	94.3 ± 0.2	91.7 ± 1.1	83.6 ± 0.5
PMP‐group					
*Proteus mirabilis*	3	99.7 ± 0.1	99.4 ± 0.1	99.8 ± 0.1	98.9 ± 0.3
*Morganella morganii*	1	100.0 ± 0	100.0 ± 0	99.3 ± 0.1	100.0 ± 0
*Providencia rettgeri*	2	98.4 ± 0	99.8 ± 0.1	99.4 ± 0.1	99.8 ± 0.2
Non‐*Enterobacteriaceae*					
PAS‐group					
*Pseudomonas aeruginosa*	3	98.6 ± 0	99.2 ± 0.0	96.2 ± 1.5	98.7 ± 0.1
*Acinetobacter baumannii*	3	97.8 ± 0	98.7 ± 0.0	98.9 ± 0.0	93.2 ± 3.0
*Staphylococcus aureus*	3	98.9 ± 0	99.6 ± 0.0	95.9 ± 1.4	99.7 ± 0.2

**a**. KECS group (*Klebsiella, Enterobacter, Citrobacter* and *Serratia* spp.) library consisted of 505 scatter images; PMP group (*Proteus, Morganella* and *Providencia* spp.) of 266 scatter images; and PAS group (*Pseudomonas, Acinetobacter* and *Staphylococcus* spp.) of 690 scatter images. **b**. Percentage average scores ± standard error of mean (SEM) values were calculated from the analysis of the colony scatter patterns acquired from two independent experiments with two replicates (*n* = 4).

### Sensitivity, specificity, PPV and negative predictive value (NPV)

A cross‐validation experiment was also performed to compute sensitivity, specificity, PPV and NPV (Baldi *et al*., 2000) for the scatter image libraries of KECS, PMP and PAS groups. The sensitivity describes the probability that our classifier will produce a true result when used on a population of colonies that contain a colony of an *Enterobacteriaceae* and non‐*Enterobacteriaceae* genera of interest. The sensitivity values for the pathogens belonging to KECS, PMP and PAS groups were very high, falling in the range of 87.1–93.2%, 98.4–100% and 97.8–98.9% respectively (Table 2). The specificity defines the probability that the test will produce a true negative result when used on colonies formed by organisms other than the pathogen of interest. The specificity for the KECS, PMP and PAS groups was in the range of 73.1–97.6%, 99.4–100% and 98.7–99.6% respectively (Table 2). The NPV is the probability that a colony does not represent a pathogen of interest when a negative result is returned. The NPVs for the KECS, PMP and PAS groups were in the range of 88.2–91.7%, 99.3–99.8% and 95.9–98.9% respectively (Table 2). The PPV gives the probability of finding positives when a BARDOT system identifies after a match with the scatter images from a library. The PPVs for the KECS, PMP and PAS groups were high and were in the range of 83.6–93.8%, 98.9–100% and 93.2–99.7% respectively (Table 2). The PPV and NPV presented in Table 2 were applied to classify each bacterial species within a group (KECS, PMP or PAS). The scatter image libraries for the KECS, PMP and PAS were used independently, and the resulting NPV and PPV do not imply for the inter‐group differentiation. These data demonstrate that using the three scatter image libraries can potentially differentiate colonies of *Enterobacteriaceae* and non‐*Enterobacteriaceae* genera, especially the species of *Klebsiella*, *Enterobacter*, *Citrobacter*, *Proteus*, *Morganella* and *Providencia* on CHROMagar^TM^ Orientation with high accuracy. The ability of group‐specific scatter image libraries to identify true positive and true negatives is described in more detail in the next section.

### Scatter pattern‐based multi‐pathogen differentiation and detection

**Figure 2:**
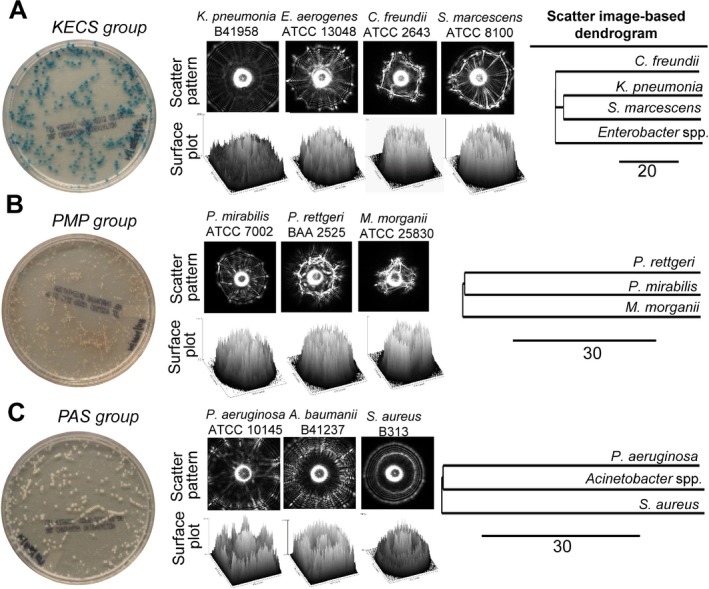
Scatter pattern‐based analysis of the *E* *nterobacteriaceae* and non‐*Enterobacteriaceae* species as a mixed culture on the CHROMagar^TM^ Orientation medium. The Petri dish on the left‐hand side shows the mixed culture colonies for the respective groups; circular images are the representative scatter pattern of single colony; three‐dimensional circular plots are the surface plots of the scatter images. The overnight (14–16 h) grown bacterial cultures (10^8^–10^9^ CFU ml^−1^) were mixed (1:1:1:1) in PBS, serially diluted, plated on CHROMagar^TM^ Orientation medium, and incubated for 10–22 h or until the colonies reached a diameter of 1.0 ± 0.2 mm. Plates were then screened by BARDOT, and scatter images were matched against the image libraries for KECS, PMP and PAS groups separately. Representative scatter images of (A) (KECS group): *Klebsiella*, *E* *nterobacter*, *C* *itrobacter* and *S* *erratia*; (B) PMP group: *P* *roteus*, *M* *organella* and *P* *rovidencia*; and non*‐* *E* *nterobacteriaceae* (C) (PAS group): *P* *seudomonas*, *A* *cinetobacter* and *S* *taphylococcus*. Representative surface plots depicting a three‐dimensional display of the intensities of pixels in a greyscale or pseudo colour image (non‐RGB images) to qualitatively visualize the differences in scatter pattern of colonies of different bacteria after circular selection of the entire scatter image using NIH imagej software and are plotted below each scatter pattern (Schneider *et al*., 2012). For greyscale values, x and y axis range were 678–992 pixels and z axis (intensity scale) was set at 0–255. Phenogram (dendrogram) was generated from the scatter patterns of three strains of each pathogen except for *P* *rovidencia rettgeri* (two strains) and *M* *. morganii* (one strain), using BioNJ algorithm (Dereeper *et al*., 2008). Dendrogram was constructed after analysing Newick output file in tree viewer (TreeDyn). All analyses were performed using open‐access algorithm and programmes available at www.phylogeny.fr. Scale bar represents distance as percentage dissimilarity.

Next we examined if these libraries could be used to differentiate colonies of different bacterial genera within the KECS, PAS and PMP group separately as mixed culture. The overnight (14–16 h) grown cultures were mixed (1:1:1:1) in PBS, serially diluted, plated on CHROMagar^TM^ Orientation medium, and incubated for 10–22 h or until the colonies reached to the desired diameter of 1.0 ± 0.2 mm. Plates were then screened by BARDOT, and scatter images were matched against the scatter image libraries for KECS, PMP and PAS groups. The representative scatter image of each species of KECS (Fig. 2A), PMP (Fig. 2B) and PAS group (Fig. 2C) revealed differential scatter features on CHROMagar^TM^ Orientation medium. Furthermore, analysis of the scatter pattern by the surface plot profile using imagej also revealed qualitative differences in the scatter pattern of *Enterobacteriaceae* in respective groups (Fig. 2A–C). The scatter pattern‐based dendrogram was also constructed from cross‐validation matrix of scatter images of KECS, PMP and PAS groups as described in our previous study (Singh *et al*., 2015a). The bacterial species within the libraries of KECS, PMP and PAS groups showed divergent branching in the dendrogram. Such divergent branching confirmed differentiating scatter patterns among the bacterial genera within KECS, PMP and PAS groups, which could not be distinguished based on the colour alone on the CHROMagar^TM^ Orientation medium (Fig. 2A–C). BARDOT also facilitated rapid identification of *E. coli* within 12–17 h (Fig. 1, Table 1), compared with colour‐based detection on CHROMagar^TM^ Orientation medium where the turnaround time was approximately 20–32 h (Manickam *et al*., 2013).

We also tested the robustness of the group‐specific libraries (KECS, PMP and PAS) by matching the scatter pattern of strains that were not included in the group‐specific libraries (Table S1). A total of 991 scatter patterns from individual strains or mixed strains were matched separately with the group‐specific libraries. The scatter pattern of colonies of *Proteus vulgaris* PRI 365, *P. vulgaris* ATCC 33420 and *P. mirabilis* ATCC 25933 that were not included in the library revealed 96%, 98% and 97% classification accuracy (PPV), respectively, with the *Proteus* genus when matched against the PMP‐Library. As anticipated, the classification accuracy for the same strains against the KECS‐Library was only 58% or less. However, the *Proteus* species also showed a high PPV (≥ 89%) when matched against the PAS‐Library (Table S1). This could be attributed to the shared scatter features between the strains of *Proteus* and *Pseudomonas* species tested. The colony scatter patterns of *Staphylococcus aureus* B41012 and *Pseudomonas fluorescens* ATCC 13525, matched against the PAS‐Library individually or in a mixture, also revealed high classification accuracy (> 96%).

For cross‐validation purposes, we also matched colony scatter patterns of *Citrobacter freundii* B2643 and *Klebsiella pneumonia* ATCC 51036 (members of the KECS group) as true negatives against the PMP and PAS libraries. As expected, this analysis revealed a very low PPV (≤ 57%). Likewise, *Proteus* species also revealed a low PPV with the KECS (≤ 58%), but high PPV (≥ 85%) when matched against the PAS‐Library (Table S1). Thus we conclude that a PPV above 90% is considered true positive, while a PPV below 90% may have the possibility of producing false‐positive result.

The food industry routinely monitors *Enterobacteriaceae* and/or the coliforms, a subgroup of *Enterobacteriaceae*, as indicator organism to assess good manufacturing practice and sanitary conditions employed during food processing (Kornacki, 2011; Buchanan and Oni, 2012; Barco *et al*., 2015). The ability of coliforms to ferment lactose is used as a marker to identify and enumerate them during conventional culturing; however, some non‐coliforms such as *Aeromonas* spp., can ferment lactose and produce false results (Baylis *et al*., 2011). Among the coliforms, *Enterobacter*, *Klebsiella*, *Citrobacter* and *Escherichia* (particularly *E. coli*) are important, and BARDOT showed high PPV (93.2–93.8%) for *Enterobacter* spp. and *Citrobacter freundii*, and slightly lower PPV for *Klebsiella pneumoniae* and *Serratia marcescens* (83.6–88.2%). These data indicate that BARDOT would be useful for analysis of both *Enterobacteriaceae* and coliforms on CHROMagar^TM^ Orientation medium (Table 2).

The laser optical sensor applied in this study is an emerging detection technology that offers a rapid, label‐free, non‐invasive, real‐time and on‐plate screening of EB on CHROMagar^TM^ Orientation medium as a companion tool. This sensor does not destroy the integrity of the colonies; thus, they can be used for further molecular and serological characterizations. To the best of our knowledge, this is the first report where a laser sensor assisted differentiation of genera within the KECS, PMP and PAS group colonies with PPVs of > 90%, when the CHROMagar^TM^ Orientation medium alone could not differentiate bacteria due to similar chromogenic properties. This technology has the potential to facilitate high‐throughput screening of colonies of *Enterobacteriaceae* for both clinical and food safety application, and could assist in making informed decision and reducing workload of laboratories in public health and food industry while detecting and differentiating multiple pathogens at the same time.

Conventional culturing method is considered a gold standard and is integral to all official detection methods (Swaminathan and Feng, 1994; FDA, 2001; Bruins *et al*., 2004; USDA‐FSIS, 2015). Moreover, a brief culturing (enrichment) is recommended for all commercially available rapid methods. This is because culturing increases target microorganism numbers, thus essential for addressing ‘zero tolerance’ policy for many food‐borne pathogens, where a single viable cell per 25 g of analytical unit can be detected with high accuracy (Swaminathan and Feng, 1994; Hahm *et al*., 2015). BARDOT fits well with the culturing method and can be used as a screening tool to isolate suspect colonies for further verification by polymerase chain reaction, next‐generation sequencing and mass‐spectrometry (Bhunia, 2014). Therefore, it has the potential to be an indispensable tool in the food testing and public health laboratories.

## Supporting information


**Table S1.** Analysing robustness of the group‐specific libraries with the scatter pattern of test strains.
