# Supplementary material for: Optical scatter patterns facilitate rapid differentiation of E nterobacteriaceae on CHROMagarTM Orientation medium
Source: Microb Biotechnol. 2015 Oct 27;9(1):127–35. doi: 10.1111/1751-7915.12323 (PMC4720409; doi:10.1111/1751-7915.12323)
Supplement: Supplementary file 1 — Table S1. Analysing robustness of the group‐specific libraries with the scatter pattern of test strains. [file MBT2-9-127-s001.docx]

**Table S1.** Analyzing robustness of the group-specific libraries with the scatter pattern of test strains.

| **Test strains** | **No. of colonies tested (*n*)** | **% Classification accuracy of test strains after matching with the scatter image library*^a^*** | | | | | | | | | |
| --- | --- | --- | --- | --- | --- | --- | --- | --- | --- | --- | --- |
|  |  | **KECS group** | | | | **PMP group** | | | **PAS group** | | |
|  |  | **K** | **E** | **C** | **S** | **P** | **M** | **P** | **P** | **A** | **S** |
| **Single strains** |  |  |  |  |  |  |  |  |  |  |  |
| *Proteus vulgaris* PRI 365 | 129 | 0 | 43 | 41 | 16 | **96** | 1 | 3 | 90 | 9 | 1 |
| *Proteus vulgaris* ATCC 33420 | 85 | 0 | 58 | 37 | 5 | **98** | 1 | 1 | 89 | 8 | 3 |
| *Proteus mirabilis* ATCC 25933 | 80 | 10 | 48 | 35 | 7 | **97** | 2 | 1 | 85 | 13 | 2 |
| *Citrobacter freundii* B2643 | 150 | 2 | 1 | **96** | 1 | 38 | 5 | 57 | 63 | 36 | 1 |
| *Klebsiella pneumonia* ATCC 51036 | 122 | **98** | 0 | 0 | 2 | 57 | 6 | 37 | 54 | 44 | 2 |
| *Staphylococcus aureus* B41012 | 235 | 31 | 46 | 23 | 0 | 47 | 10 | 43 | 0 | 2 | **98** |
| *Pseudomonas fluorescens* ATCC 13525 | 90 | 41 | 33 | 20 | 6 | 80 | 5 | 15 | **99** | 1 | 0 |
| **Mixed strains*^b^*** |  |  |  |  |  |  |  |  |  |  |  |
| *Staphylococcus aureus* B41012 | 50 | ------------------------Not tested-------------------- | | | | | | | 1 | 2 | **97** |
| *Pseudomonas fluorescens* ATCC 13525 | 50 | ------------------------Not tested-------------------- | | | | | | | **98** | 2 | 0 |
| Total colonies tested | 991 |  |  |  |  |  |  |  |  |  |  |

*^a^*Test samples from single strains were matched separately with the scatter image library of KECS group [species of *Klebsiella* (**K**), *Enterobacter* (**E**), *Citrobacter* (**C**), and *Serratia* (**S**)]; PMP group [species of *Proteus* (**P**), *Morganella* (**M**), and *Providencia* (**P**)]; PAS group [species of *Pseudomonas* (**P**), *Acinetobacter* (**A**), and *Staphylococcus* (**S**)].

*^b^*More than 100 colonies were screened with the BARDOT from duplicate plates, and colonies with *Staphyloccous*-type scatter pattern (concentric rings without spokes and colonies with *Pseudomonas*-type scatter pattern (radial overlapping spokes) were also matched with the PAS Library; and results are presented as % classification accuracy also known as % positive predictive value (PPV).

Test strains that showed high classification accuracy or PPV (>90%) and are correctly classified with their respective class are highlighted in bold.
